# Supplementary material for: A liar and a copycat: nonverbal coordination increases with lie difficulty
Source: R Soc Open Sci. 2021 Jan 13;8(1):200839. doi: 10.1098/rsos.200839 (PMC7890472; doi:10.1098/rsos.200839)

**Supplementary Materials**

This document provides supplementary information about the Procedure used in the paper “A Liar and a Copycat: Nonverbal Mimicry Increases with Lie Difficulty.”

Interview instructions

The experimenter gave the following account to encourage the participant to deceive in each condition.

**Easy lie.** “Sorry, that’s my fault. I do not think it’s really a problem for the experiment. It’s just that I started my PhD here recently and my supervisor is going to teach me how to code the interview videos. Would you mind not mentioning seeing the instructions when you get asked about them in the interview?”

**Difficult and very difficult lie.** “For this part of the task you are going to have to use your imagination by making up a story about playing a game of Cluedo (also known as Clue) with three other players. The other participant has just gone to meet these three other players but the game is a set up. The three players are confederates who will act in certain ways that move the game to a predetermined outcome. The interviewer has information about this game and his task is to try and work out whether the other participant or you were the fourth player in the game. So, your task is to convince the interviewer that you played Cluedo with the three confederates. If you do this successfully, you will be entered into a prize draw to win an iPod.”

WiTilt Device Placement

Figure SM1 gives an example of the Wi-Tilt placement on each participant (provided with participant consent). The box-file in front of the interviewer (left panel) housed the questions in a way that obscured them from view of the interviewee.

Figure SM1. Interviewer (left) and interviewee (right) during an interview. Both participants are wearing four WiTilts to measure mimicry occurrence.


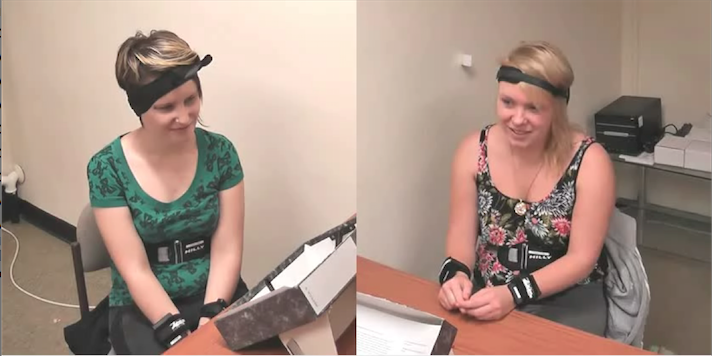

Supplement: Supplementary Materials [file rsos200839supp1.docx]
